# Evolution of Untreated Moderate Mitral Regurgitation After Transcatheter Aortic Valve Implantation

**DOI:** 10.3390/medicina61040686

**Published:** 2025-04-09

**Authors:** Massimo Baudo, Serge Sicouri, Francesco Cabrucci, Yoshiyuki Yamashita, Dimitrios E. Magouliotis, Sarah M. Carnila, Sandra V. Abramson, Katie M. Hawthorne, Harish Jarrett, Roberto Rodriguez, Scott M. Goldman, Paul M. Coady, Eric M. Gnall, William A. Gray, Sandro Gelsomino, Basel Ramlawi

**Affiliations:** 1 Department of Cardiac Surgery Research, Lankenau Institute for Medical Research, Main Line Health, Wynnewood, PA 19096, USA; sicouris@mlhs.org (S.S.); francesco.cabrucci.6@gmail.com (F.C.); yamashitay@mlhs.org (Y.Y.); magouliotisd@mlhs.org (D.E.M.); sarahmaggie14@gmail.com (S.M.C.); grayw@mlhs.org (W.A.G.); ramlawib@mlhs.org (B.R.); 2 Department of Cardiac Surgery, Lankenau Heart Institute, Main Line Health, Wynnewood, PA 19096, USA; rodriguezr@mlhs.org (R.R.); goldmans@mlhs.org (S.M.G.); 3 Department of Interventional Cardiology, Cardiovascular Imaging Center, Lankenau Heart Institute, Main Line Health, Wynnewood, PA 19096, USA; abramsons@mlhs.org (S.V.A.); hawthornek@mlhs.org (K.M.H.); jarretha@mlhs.org (H.J.); 4 Department of Interventional Cardiology, Lankenau Heart Institute, Main Line Health, Wynnewood, PA 19096, USA; coadypa@mlhs.org (P.M.C.); gnalle@mlhs.org (E.M.G.); 5 Cardiovascular Research Institute Maastricht—CARIM, Maastricht University Medical Centre, 6229 ER Maastricht, The Netherlands; sandro.gelsomino@maastrichtuniversity.nl

**Keywords:** TAVI, transcatheter aortic valve intervention, mitral regurgitation

## Abstract

*Background and Objectives*: Associated mitral regurgitation (MR) is frequently observed during transcatheter aortic valve implantation (TAVI). The progression of moderate MR remains undetermined, given uncertain clinical significance and natural history. This study aims to assess the evolution of moderate MR following TAVI. *Materials and Methods*: Between 2018 and 2023, 1476 patients underwent TAVI. We excluded those with previous aortic or mitral valve interventions, endocarditis, concomitant percutaneous coronary intervention, or emergent procedures. Patients with severe aortic or tricuspid regurgitation or significant mitral stenosis were excluded. Ultimately, only patients with moderate MR were included, resulting in a final population of 154 patients. *Results*: Mean age was 81.4 ± 7.8 years, 48.1% (74/154) were female, and 48.1% (74/154) were functional MR. There was one surgical conversion due to annular rupture. Thirty-day mortality was 1.9% (3/154). Postoperative echocardiography showed 38 (24.7%) patients with none/trace MR, 91 (59.1%) with mild MR, 22 (14.3%) with moderate MR, and 3 (1.9%) with severe MR. Finally, according to the echocardiographic follow-up [median follow-up 1.0 (IQR: 0.1–1.2) years], 20.1% (31/154) had no/trace MR, 39.6% (61/154) had mild MR, 35.7% (55/154) had moderate MR, and 4.5% (7/154) had severe MR. Overall, 67 (43.5%) patients had any MR grade progression, 62 (40.3%) had stable disease, and 25 (16.2%) had any MR grade reduction at the last follow-up from the operation. No difference in MR evolution was seen between functional and primary MR. *Conclusions*: Concomitant moderate MR during TAVI has a variable evolution over time. A more detailed characterization of patients with preoperative moderate MR undergoing TAVI is necessary to identify those with a disease progression risk.

## 1. Introduction

Patients undergoing transcatheter aortic valve implantation (TAVI) frequently present with coexisting mitral regurgitation (MR), with rates reported as high as 15% to 25% prior to the intervention [1,2,3,4]. In many cases, MR is secondary to abnormal left ventricular function and annular dilatation (functional MR, fMR), which result from a chronically elevated afterload [3]. As a result, MR often improves after TAVI due to favorable ventricular remodeling in patients with severe aortic stenosis (AS) [1,4]. However, the interaction between AS and MR presents a unique diagnostic challenge. AS can lead to an overestimation of MR severity due to increased end-systolic pressure, while MR’s reduced forward ejection fraction can lead to an underestimation of AS severity, potentially misclassifying classic severe AS as low-gradient AS [5]. This diagnostic ambiguity complicates treatment decisions.

In cases of severe AS and severe MR, combined valve surgery is recommended to improve survival [6,7]. However, when moderate MR is secondary to severe AS, the optimal timing and treatment strategy remain uncertain, with current guidelines generally leaving the decision to the heart team. While severe or moderate-to-severe MR (>moderate) is typically addressed surgically during aortic valve replacement, and none/trace or mild MR (<moderate) is left untreated, the management of concomitant moderate MR remains unclear. This is due to the uncertainty surrounding its clinical significance and natural regurgitation progression. In patients undergoing TAVI, this issue becomes even more complex, as they are typically at a higher risk, and addressing concomitant pathologies exponentially increases the overall risk. In the era of surgical management, combined AS and MR could be treated using the same procedure, albeit with increased operative mortality compared to single-valve surgery [8]. Indeed, multivalvular diseases such as AS and MR are strongly associated with aging, and patients may not be candidates for combined surgical treatment due to high surgical risks. Some studies have demonstrated an independent association between moderate/severe MR and increased mortality [9,10,11,12], while others showed no significant difference in mortality at follow-up [13,14].

Another issue regards the identification of possible predictors of low MR regression after TAVI, as only a few have been identified so far, including mitral annular and leaflet calcification, along with annular dilatation [15]. Patients with primary MR tend to have lower rates of MR improvement and higher mortality compared to those with functional MR, particularly atrial fMR [16].

The aim of this study was to evaluate the early and mid-term fate of moderate MR in patients undergoing TAVI for severe AS. The progression or regression of preoperative moderate MR will be monitored from the postoperative stay through follow-up, and the possible impact on mortality and clinical outcomes will be evaluated.

## 2. Materials and Methods

Patients who underwent TAVI for severe native valve AS at Lankenau Heart Institute in Wynnewood, PA, USA, between January 2018 and December 2023 were retrospectively included in this study. The purpose of the study was to evaluate the progression of associated moderate MR after TAVI. The study protocol received approval from the Institutional Review Board of Main Line Health Hospitals (IRB 45CFR164.512), and patient consent was waived due to the study’s retrospective design.

Clinical and procedural data were collected from medical records. All patients underwent pre-procedural transthoracic and/or transesophageal echocardiography, as well as gated computed tomography angiography with multiphase reconstruction for TAVI evaluation.

### 2.1. Inclusion Criteria

Eligible patients had severe native valve AS, defined by an aortic valve area ≤ 1.0 cm^2^ (or an indexed aortic valve area ≤ 0.6 cm^2^/m^2^), a mean gradient of ≥40 mmHg, or a peak aortic valve velocity of ≥4.0 m/s on resting transthoracic echocardiography. Exclusion criteria included any prior aortic or mitral valve intervention, with the primary indication for TAVI being aortic regurgitation or endocarditis, concomitant percutaneous coronary intervention, or n emergent procedure. Patients with severe aortic or tricuspid regurgitation or significant mitral stenosis (mean gradient > 5 mmHg) were also excluded.

### 2.2. Statistical Analysis

Categorical variables were reported as frequencies and percentages. Continuous variables were tested for normal distribution using the Kolmogorov–Smirnov test, with normally distributed data presented as mean and standard deviation, and non-normally distributed data reported as median and interquartile range (IQR). Survival analysis was illustrated using Kaplan–Meier curves, displaying estimates along with their standard errors. Univariable Cox regression analysis evaluated the impact of MR progression on the clinical follow-up data.

The change in NYHA functional class, whether improved or worsened, was analyzed by comparing values from the pre-operative period to the 30-day evaluation. This change, defined as a shift from the baseline value, was assessed using symmetry tests, specifically McNemar–Bowker’s test with the Bonferroni method for multiple *p*-values adjustment, and Bhapkar’s test. McNemar–Bowker’s test checks for symmetry, while the Bhapkar test examines marginal homogeneity. When both tests produce significant *p*-values, it indicates a meaningful shift.

A Sankey diagram was used to visually represent the changes in mitral valve regurgitation across the preoperative, postoperative and follow-up stages.

The impact of preoperative BNP on MR progression, moderate/severe MR, death and rehospitalization for HF was evaluated with logistic regression analysis. Results were presented as an estimate (β) ± standard error.

All statistical tests were two-sided, with a significance level of 0.05. Statistical analysis was conducted using R version 4.4.1 (R Project for Statistical Computing, Vienna, Austria) in RStudio (Version 2024.12.1+563). Data supporting the study’s findings can be made available upon reasonable request, subject to institutional approval. The study adhered to the STROBE (STrengthening the Reporting of OBservational Studies in Epidemiology) guidelines for reporting observational studies [17].

## 3. Results

Between 2018 and 2023, a total of 1476 TAVI procedures were carried out at our center. For this analysis, 159 patients were excluded due to previous aortic and/or mitral valve interventions, 22 had simultaneous PCI, 5 were excluded due to endocarditis, and 3 required emergency interventions. Furthermore, 18 and 33 patients were excluded for severe aortic and tricuspid regurgitation, respectively. One thousand seventy patients had an MR other than moderate, and sixteen had a mitral stenosis >5 mmHg. The final population consisted of 154 patients. The median clinical follow-up was 2.0 (interquartile range [IQR]: 1.1–3.2) years, while the median echocardiographic follow-up was 1.0 (IQR: 0.1–1.2) years.

The baseline characteristics and preoperative echocardiographic assessments are summarized in Table 1. The mean age was 81.4 ± 7.8 years, and 48.1% (*n* = 74) were female. Ninety-five (61.7%) patients were classified as NYHA class III-IV. Computed tomography revealed a mean valve area of 502 ± 107 mm^2^ and a mean perimeter of 81.0 ± 8.8 mm. Preoperative echocardiography indicated a mean aortic valve area of 0.71 ± 0.18 cm^2^, with a median ejection fraction of 58.0% (IQR: 40.8–65.0). FMR was present in 74 cases (48.1%).

Patients were evenly divided between receiving balloon-expandable (*n* = 71, 51.4%) and self-expandable valves (*n* = 67, 48.6%). There was only one case of surgical conversion (0.6%), which resulted from intraoperative annular rupture. The patient required a median sternotomy to control bleeding, which had led to hemodynamic instability due to cardiac tamponade. Hemostasis was achieved by using interrupted sutures, and the patient was discharged four days later. The median length of hospital stay was 1.0 days (IQR: 1.0–3.0), and the 30-day mortality rate was 1.9% (*n* = 3). Perioperative outcomes are detailed in Table 2. In most cases (*n* = 129, 83.7%), mitral regurgitation improved postoperatively to none/trace or mild, remained stable in 22 patients (14.3%), and worsened in 3 patients (1.9%) (fMR, *n* = 1).

The 30-day NYHA functional class was available for 89.0% of patients (137 out of 154). A significant improvement in functional class was observed compared to the preoperative status, as demonstrated by concomitant Bhapkar’s test (*p* < 0.001) and McNemar–Bower test (*p* < 0.001) significance; Table S1.

**Figure 1:**
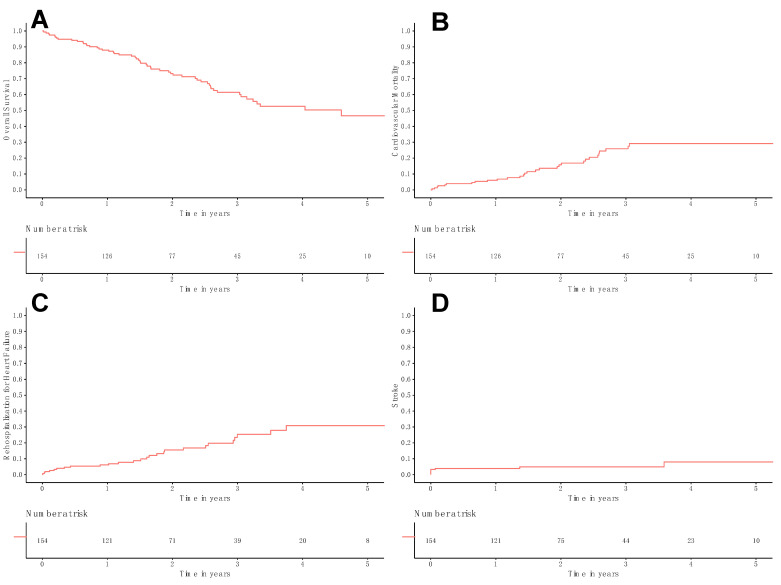
Kaplan–Meier curves of follow-up outcomes. The population is depicted with a red line. Kaplan–Meier curves for: (**A**) Overall survival, (**B**) cardiovascular-related mortality, (**C**) rehospitalization for heart failure, (**D**) stroke.

The overall survival rates at 1, 3, and 5 years were 88.0% ± 2.7%, 61.4% ± 4.7%, and 46.7% ± 6.1%, respectively (Figure 1A). Cardiovascular-related mortality at 1, 3, and 5 years was 6.1% ± 2.0%, 25.9% ± 4.5%, and 29.2% ± 4.9% (Figure 1B). The rates of rehospitalization for heart failure at 1, 3, and 5 years were 6.1% ± 2.0%, 25.3% ± 4.9%, and 30.8% ± 5.9% (Figure 1C). Stroke rates at 1, 3, and 5 years were 3.9% ± 1.6%, 4.9% ± 1.8%, and 8.0% ± 3.5%, respectively (Figure 1D). During the follow-up period, three patients required mitral valve procedures. Two patients underwent MitraClip interventions, occurring 0.9 and 2.3 years after the TAVI procedure, while one patient received a transcatheter mitral valve replacement 2.4 years post-TAVI.

At univariable Cox regression MR progression was not associated with overall survival (HR: 1.06, 95% CI: 0.62–1.81, *p* = 0.837, Figure S1), rehospitalization for heart failure (HR: 1.46, 95% CI: 0.68–3.17, *p* = 0.334, Figure S2), cardiovascular mortality (HR: 1.31, 95% CI: 0.63–2.72, *p* = 0.466, Figure S3), or stroke (HR: 1.42, 95% CI: 0.35–5.72, *p* = 0.621, Figure S4).

**Figure 2:**
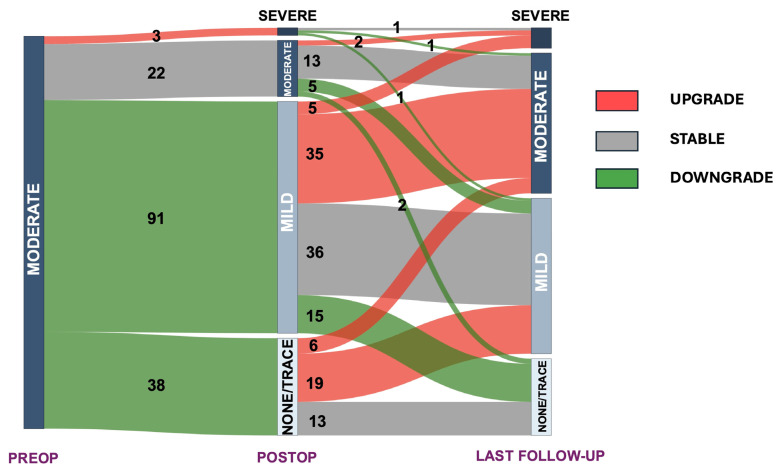
Sankey plot of mitral regurgitation progression from preoperative (moderate) to last echocardiographic follow-up. Numbers represent number of patients and mitral regurgitation evolution is color-coded: red = upgrade; gray = stable disease; green = downgrade.

**Figure 3:**
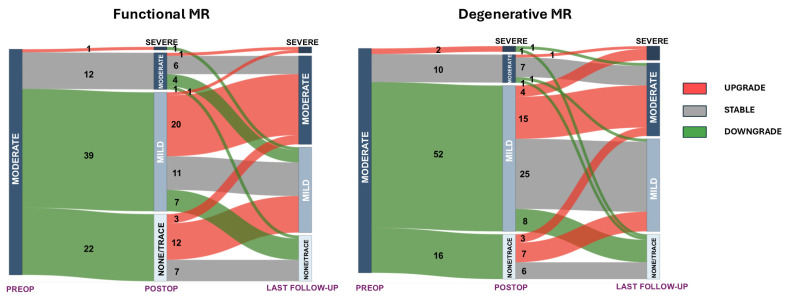
Sankey plot of mitral regurgitation progression from preoperative (moderate) to last echocardiographic follow-up by etiology. Numbers represent number of patients and mitral regurgitation evolution is color-coded: red = upgrade; gray = stable disease; green = downgrade.

At the latest follow-up echocardiogram, 92 patients (59.7%) had less than moderate MR, 35.7% had moderate MR (*n* = 55), and 7 patients (4.5%) had severe MR (2 were fMR) (Table 3). Overall, 67 (43.5%) patients had any form of MR grade progression, 62 (40.3%) had any form of MR grade stable disease, and 25 (16.2%) had any form of MR grade reduction at the last follow-up from discharge. The overall MR evolution from preoperative moderate MR can be seen on the Sankey plot in Figure 2. The separate progression of degenerative MR (dMR) and fMR is depicted in Figure 3. For fMR, 17.6% (13/74) of patients had any form of MR grade reduction, 32.4% (24/74) had any form of MR-grade stable disease, and 50.0% (37/74) had any form of MR-grade progression at the last follow-up from the discharge. On the other hand, for dMR patients, 15.0% (12/80) of patients had any form of MR grade reduction, 47.5% (38/80) had any form of MR-grade stable disease, and 37.5% (30/80) had any form of MR-grade progression at last follow-up from the discharge. No significant difference in this regard was reported between the two groups (*p* = 0.157).

Patients with a postoperative MR ≥moderate showed similar rates of rehospitalization for heart failure at follow-up when compared to MR <moderate (12/62, 19.4% vs. 14/92, 15.2%, *p* = 0.501). Similar mortality follow-up rates were reported for patients with a postoperative MR ≥moderate compared to those with an MR <moderate (24/62, 38.7% vs. 31/92, 33.7%, *p* = 0.524).

At regression analysis preoperative BNP was significantly and positively associated with MR progression (9.231 × 10^−5^ ± 4.181 × 10^−5^, *p* = 0.029), last follow-up moderate/severe MR (1.021 × 10^−5^ ± 4.122 × 10^−5^, *p* = 0.014) and mortality (9.964 × 10^−5^ ± 4.030 × 10^−5^, *p* = 0.015), but not rehospitalization for HF (−6.847 × 10^−5^ ± 3.221 × 10^−5^, *p* = 0.832).

## 4. Discussion

In this study, we assessed the progression of moderate MR following TAVI and observed the following: (1) there was a variable response post-procedure, with the majority of patients (*n* = 129, 83.7%) experiencing a reduction in mitral insufficiency to less than moderate MR and a symptomatic benefit overall; (2) however, at follow-up, the proportion of patients with ≥moderate MR increased (*n* = 62, 40.3%) compared to the postoperative evaluation; (3) MR progression was not associated with clinical follow-up endpoints; (4) in the end, only 4.5% (*n* = 7) of patients had severe MR from the preoperative moderate MR; (5) the group with ≥moderate MR had similar mortality and rehospitalization for heart failure rates compared to those with <moderate MR, which could possibly explained by the minimal persistence of an advanced NYHA class.

TAVI often results in an improvement in preoperative MR severity after the procedure, a factor that correlates with better clinical outcomes [5,15,18,19], and our study is consistent with these findings, demonstrating that MR improves after TAVI in most cases (>80%). Reports on survival outcomes in patients with concomitant AS and MR have not been consistent making it critical to accurately characterize results in this relatively understudied group, especially as the use of TAVI continues to expand. On one hand, moderate to severe MR was associated with higher short-term and long-term mortality after TAVI, as observed in multiple studies and meta-analyses [1,4,5,9,10,11,12,16,20,21]; however, other studies have shown no significant difference [13,14,15]. Witberg et al. have clearly shown that mortality appears to be more closely associated with how MR responds to TAVI (post-TAVI MR grade) rather than the severity of MR before the procedure [5]. In this regard, we were unable in our study to show that MR regression had a significant benefit in terms of survival, rehospitalization for heart failure, or stroke during follow-up, possibly because the study was underpowered to assess these outcomes due to the limited number of events and the reduced sample size.

Current data show limited ability to predict MR response after TAVI due to the complex nature of MR’s underlying mechanisms [5,15,22]. Accurate prediction is critical, as it could guide treatment decisions between TAVI and double-valve surgery. So far, factors such as atrial fibrillation [15], ischemic cardiomyopathy [23,24], pre-existing aortic insufficiency [21,25], increased pulmonary artery systolic pressure [11,26,27], lower pre-TAVI mean aortic transvalvular pressure gradient [28], and left atrial dilation [15] have all been associated. Cortés et al. reported mitral annular and leaflet calcification together with mitral annular dilatation (>35.5 mm) as independent predictors of MR after TAVI [15]. The presence of mitral annular calcification with restriction was also previously described as a predictor of poor MR improvement by Durst and colleagues [22]. This partially explains why patients with primary MR have lower rates of MR improvement and higher mortality when MR persists after TAVI, compared to patients with fMR, especially in cases of atrial fMR. Doldi et al. demonstrated that MR improvement rates were significantly higher in patients with atrial fMR (80.2%) than in those with ventricular fMR (69.4%) and primary MR (40.8%) [16]. Notably, the improvement in fMR is particularly pronounced with the postoperative improvement of a reduced preoperative ejection fraction [29,30]. Although the rate of disease progression in our study was higher for dMR than fMR, the difference was not statistically significant, possibly because of the reduced sample size and a not-meaningful impaired left ventricular ejection fraction.

On the other hand, this study has shown how higher levels of preoperative BNP were significantly associated with MR progression, last follow-up moderate/severe MR and mortality. In patients with moderate mixed aortic valve disease, high BNP levels were associated with adverse clinical outcomes, suggesting that BNP could serve as a marker for overall cardiac health in these patients [31]. However, a word of caution is warranted, as the elevation of BNP may reflect underlying myocardial dysfunction rather than directly contributing to MR progression or mortality. Other confounding factors might drive both BNP elevation and MR worsening. In addition, BNP levels fluctuate based on volume status, renal function and acute decompensations. A single preoperative BNP measurement may not fully capture a patient’s long-term cardiac status.

The study by Witberg et al. showed that patients with persistent NYHA class III-IV symptoms after TAVI at 30 days were more likely to have persistent MR, which contributed to higher mortality in this group [5]. Therefore, postoperative symptom resolution despite persistent MR suggests that MR plays a lesser role in morbidity for some patients and has a lesser impact on post-TAVI outcomes. This likely helps explain why in the present study no survival difference was observed between the ≥moderate MR and <moderate MR groups, as only three patients with postoperative ≥moderate MR had an NYHA class of three or higher at 1 month.

A key unresolved aspect in determining the best treatment approach for patients with combined AS and MR is the role of staged percutaneous mitral valve repair following TAVR. Only a few patients with persistent MR undergo staged transcatheter repair, likely because patients still experience significant symptomatic relief after TAVR alone and due to unfeasibility anatomical limitations [5]. Indeed, only three patients in this analysis needed a mitral valve intervention at follow-up. However, the available data on this subject remain limited, with significant advances occurring only in recent years due to the increasing adoption of transcatheter mitral therapies. This progress could make staged transcatheter interventions more accessible to a broader patient population with persistent MR after TAVR.

Therefore, the variability in MR response underscores the need for individualized post-TAVI surveillance, incorporating early echocardiographic reassessment and clinical evaluation. Patients who continue to experience symptoms despite successful treatment of AS should be reassessed for early post-TAVR for MR severity and NYHA functional class. In selected cases, persistent MR may warrant additional therapeutic interventions, including medical therapy optimization or transcatheter mitral valve repair. However, further research is essential to develop clear guidelines for the management of patients with concurrent AS and MR.

### Limitation

The primary limitations of this study stem from its retrospective design, as there may have been confounding factors that were not accounted for. Another important consideration is that the study was conducted at a single center, which may limit the generalizability of the results to a larger population. This limitation also applies to the need for long-term follow-up, as it could uncover significant late survival differences. Moreover, the study’s average age was over 80 years, focusing mainly on patients with low/intermediate surgical risk. This could be viewed as a limitation, as younger populations with lower surgical risk might show different outcomes. Finally, the accuracy of echocardiography is highly dependent on the skill and experience of the sonographer and the interpreting physician. Variability between operators may lead to differences in image acquisition and interpretation.

## 5. Conclusions

Concomitant moderate MR during TAVI has a variable evolution, and predictors of MR regression should be further studied for optimized patient treatment. Therefore, a more detailed characterization of patients with preoperative moderate MR undergoing TAVI is needed to identify those at risk for disease progression.

## Figures and Tables

**Table 1:** Baseline patient’s demographics.

Characteristic	*n* = 154
Female sex, *n* (%)	74 (48.1)
Age, years, mean (SD)	81.4 (7.8)
BSA, m^2^, mean (SD)	1.84 (0.23)
BMI, kg/m^2^, median [IQR]	25.31 [22.54, 29.26]
NYHA functional class, *n* (%)	
Class I	5 (3.2)
Class II	54 (35.1)
Class III	73 (47.4)
Class IV	22 (14.3)
BNP, pg/mL, median [IQR]	465 [231, 998.5]
Urgent, *n* (%)	22 (14.3)
STS PROM, median [IQR]	3.60 [2.64, 5.90]
Hypertension, *n* (%)	138 (89.6)
Dyslipidemia, *n* (%)	137 (89.0)
Diabetes mellitus, *n* (%)	54 (35.1)
Liver disease, *n* (%)	5 (3.2)
eGFR, mL/min/1.73 m^2^, mean (SD)	56.79 (21.99)
Dialysis, *n* (%)	5 (3.2)
Smoke status, *n* (%)	
Active	8 (5.2)
Former	32 (20.8)
Never	114 (74.0)
Chronic lung disease, *n* (%)	22 (14.3)
Prior CVA, *n* (%)	17 (11.0)
PAD, *n* (%)	40 (26.0)
Prior MI, *n* (%)	31 (20.1)
Prior CABG, *n* (%)	28 (18.2)
Prior PCI, *n* (%)	57 (37.0)
Atrial fibrillation, *n* (%)	
No	84 (54.5)
Paroxysmal	43 (27.9)
Persistent	26 (16.9)
Long-standing Persistent	1 (0.6)
Prior pacemaker, *n* (%)	21 (13.6)
Prior ICD, *n* (%)	6 (3.9)
**MDCT**	
SoV diameter, mm, mean (SD)	33.1 (4.0)
STJ diameter, mm, mean (SD)	27.1 (4.0)
Annulus diameter, mm, mean (SD)	25.1 (3.1)
Area, mm^2^, mean (SD)	502 (107)
Perimeter, mm, mean (SD)	81.0 (8.8)
**Echocardiography**	
Ejection fraction, %, median [IQR]	58.0 [40.8, 65.0]
AVA, cm, mean (SD)	0.71 (0.18)
Indexed AVA, cm/m^2^, mean (SD)	0.39 (0.11)
Mean gradient, mmHg, mean (SD)	41.4 (13.2)
Peak velocity, m/s, mean (SD)	4.10 (0.66)

AVA = aortic valve area; BMI = body mass index; BNP = brain-derived natriuretic peptide; BSA = body surface area; CABG = coronary artery bypass graft; CVA = cerebrovascular accident; eGFR = estimated glomerular filtration rate; ICD = implantable cardioverter defibrillator; IQR = interquartile range; MDCT = multi-detector computer tomography; MI = myocardial infarction; NYHA = New York Heart Association; PAD = peripheral artery disease; PCI = percutaneous coronary intervention; SD = standard deviation; SoV = sinus of Valsalva; STJ = sinotubular junction; STS PROM = Society of Thoracic Surgeons predicted risk of mortality.

**Table 2:** Perioperative outcomes.

Outcome	*n* = 154
Transfemoral approach, *n* (%)	141 (91.6)
Pre dilatation, *n* (%)	49 (31.8)
Post dilatation, *n* (%)	10 (6.5)
Device, *n* (%)	
Evolut FX	10 (6.5)
Evolut PRO	38 (24.7)
Evolut PRO Plus	25 (16.2)
AcurateNeo 2	1 (0.6)
SAPIEN 3	55 (35.7)
SAPIEN 3 Ultra	25 (16.2)
Device size, *n* (%)	
23	18 (11.7)
26	61 (39.6)
29	63 (40.9)
34	11 (7.1)
XL	1 (0.6)
Surgical conversion, *n* (%)	1 (0.6)
Intraoperative transfusion, *n* (%)	8 (5.2)
Valve malposition, *n* (%)	0 (0.0)
Second valve, *n* (%)	0 (0.0)
Annulus rupture, *n* (%)	1 (0.6)
Cardiac tamponade, *n* (%)	1 (0.6)
Coronary obstruction, *n* (%)	0 (0.0)
Overt bleeding, *n* (%)	4 (2.6)
Major vascular complication, *n* (%)	3 (1.9)
Minor vascular complication, *n* (%)	7 (4.5)
Stroke, *n* (%)	5 (3.2)
TIA, *n* (%)	1 (0.6)
Myocardial infarction, *n* (%)	1 (0.6)
AKI >stage 2, *n* (%)	4 (2.6)
New dialysis, *n* (%)	1 (0.6)
New atrial fibrillation, *n* (%)	7 (4.5)
New pacemaker, *n* (%)	21 (13.6)
New LBBB, *n* (%)	29 (18.8)
Postop transfusion, *n* (%)	15 (9.7)
Length of stay, days, median [IQR]	1.0 [1.0, 3.0]
30-day mortality, *n* (%)	3 (1.9)
**Echocardiography**	
Ejection fraction, %, mean (SD)	57.35 (13.93)
EOA, cm^2^, median [IQR]	2.13 (0.52)
Indexed EOA, cm^2^, median [IQR]	1.18 (0.33)
Mean gradient, mmHg, mean (SD)	9.22 (4.17)
Peak velocity, m/s, mean (SD)	1.98 (0.46)
Mitral regurgitation, *n* (%)	
None/Trace	38 (24.7)
Mild	91 (59.1)
Moderate	22 (14.3)
Severe	3 (1.9)

AKI = acute kidney injury; EOA = effective orifice area; IQR = interquartile range; LBBB = left bundle branch block; MCS = mechanical circulatory support; SD = standard deviation; TIA = transient ischemic attack.

**Table 3:** Follow-up echocardiographic mitral regurgitation degree.

Follow-Up Echocardiography *	*n* = 154
Ejection fraction, %, mean (SD)	57.5 (12.5)
Mitral regurgitation, *n* (%)	
None/Trace	31 (20.1)
Mild	61 (39.6)
Moderate	55 (35.7)
Severe	7 (4.5)

* Latest FUP echo available. In the case of MV intervention, the last echo MR before intervention was used. SD = standard deviation.

## Data Availability

Data supporting the study’s findings can be made available upon reasonable request, subject to institutional approval.

## Supplementary Materials

The following supporting information can be downloaded at https://www.mdpi.com/article/10.3390/medicina61040686/s1: Table S1: Preoperative and 30-day NYHA class distribution; Figure S1—Kaplan–Meier curve for overall survival with Cox regression analysis on mitral regurgitation progression; Figure S2—Kaplan–Meier curve for rehospitalization for heart failure with Cox regression analysis on mitral regurgitation progression; Figure S3—Kaplan–Meier curve for cardiovascular mortality with Cox regression analysis on mitral regurgitation progression; Figure S4—Kaplan–Meier curve for stroke with Cox regression analysis on mitral regurgitation progression.

## Abbreviations

The following abbreviations are used in this manuscript:

AS	Aortic stenosis
dMR	Degenerative mitral regurgitation
fMR	Functional mitral regurgitation
MR	Mitral regurgitation
TAVI	Transcatheter aortic valve implantation
