# Supplementary material for: Evolution of Untreated Moderate Mitral Regurgitation After Transcatheter Aortic Valve Implantation
Source: Medicina (Kaunas). 2025 Apr 9;61(4):686. doi: 10.3390/medicina61040686 (PMC12028619; doi:10.3390/medicina61040686)

Table S1 – Preoperative and 30-day NYHA class distribution

NYHA	1	2	3	4	30-day
1	2	2	0	0	
2	38	9	4	0	
3	46	10	6	0	
4	12	7	1	0	
Preoperative					

NYHA = New York Heart Association

Figure S1 – Kaplan Meier curve for overall survival with Cox regression analysis on mitral regurgitation progression

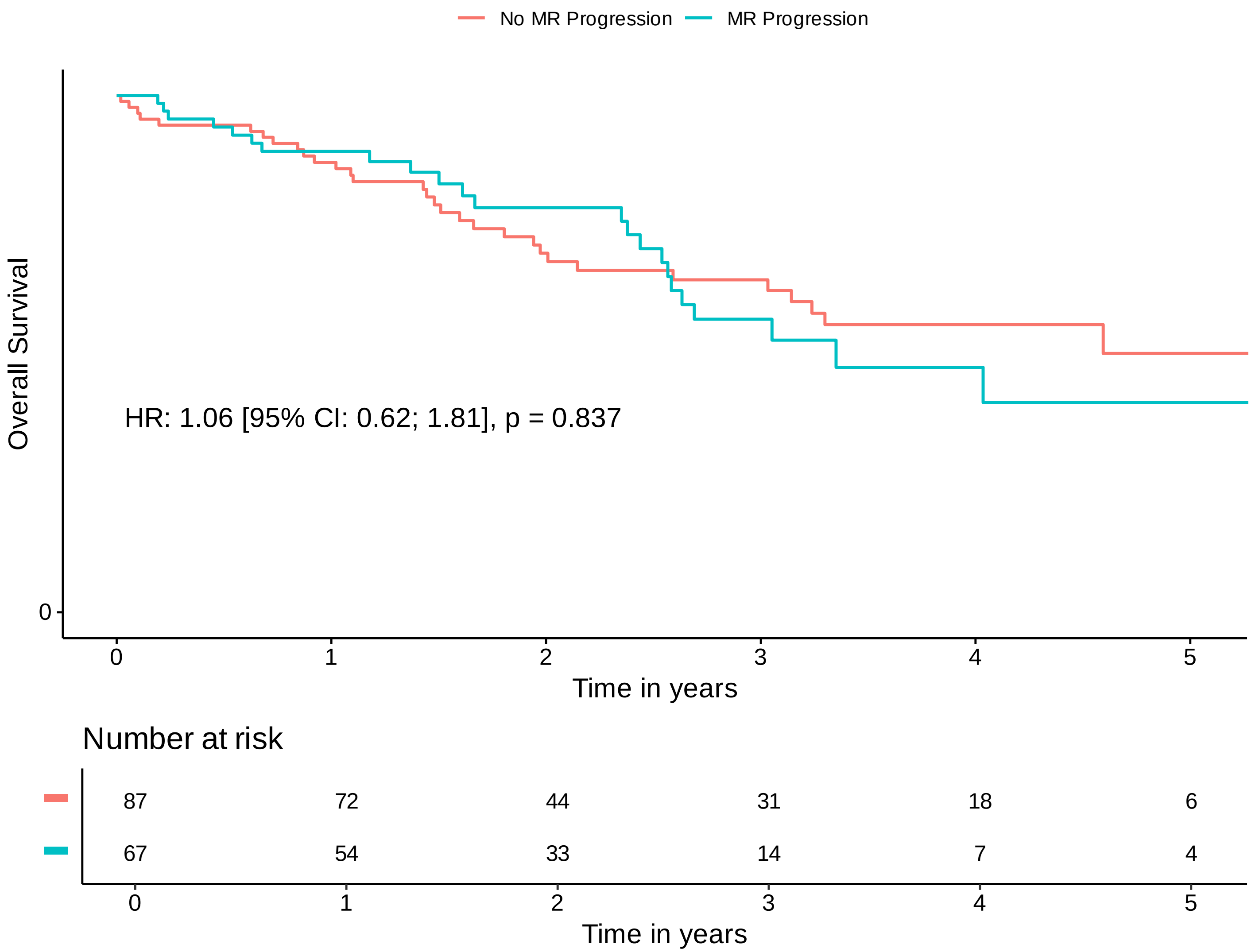

Figure S2 – Kaplan Meier curve for rehospitalization for heart failure with Cox regression analysis on mitral regurgitation progression

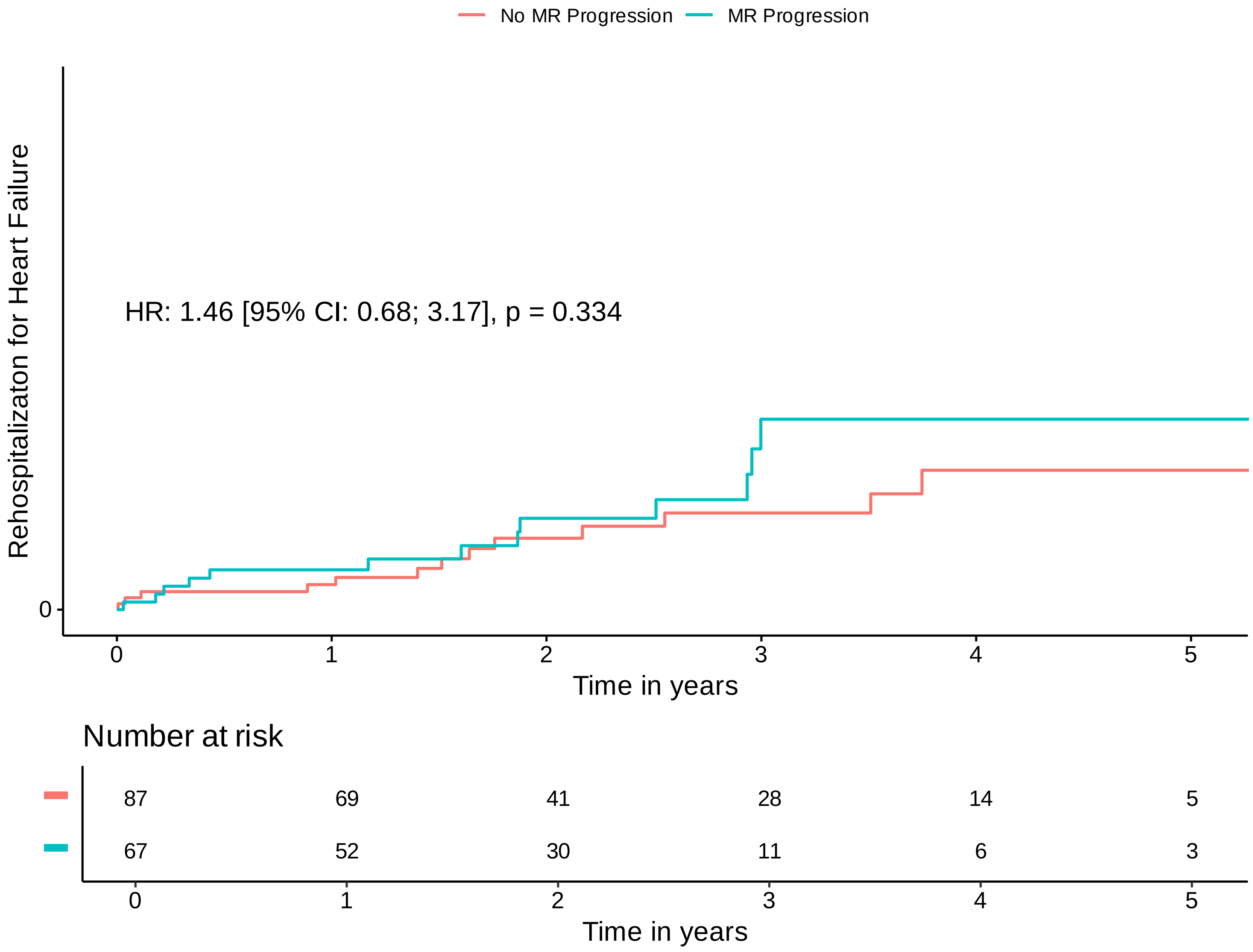

Figure S3 – Kaplan Meier curve for cardiovascular mortality with Cox regression analysis on mitral regurgitation progression

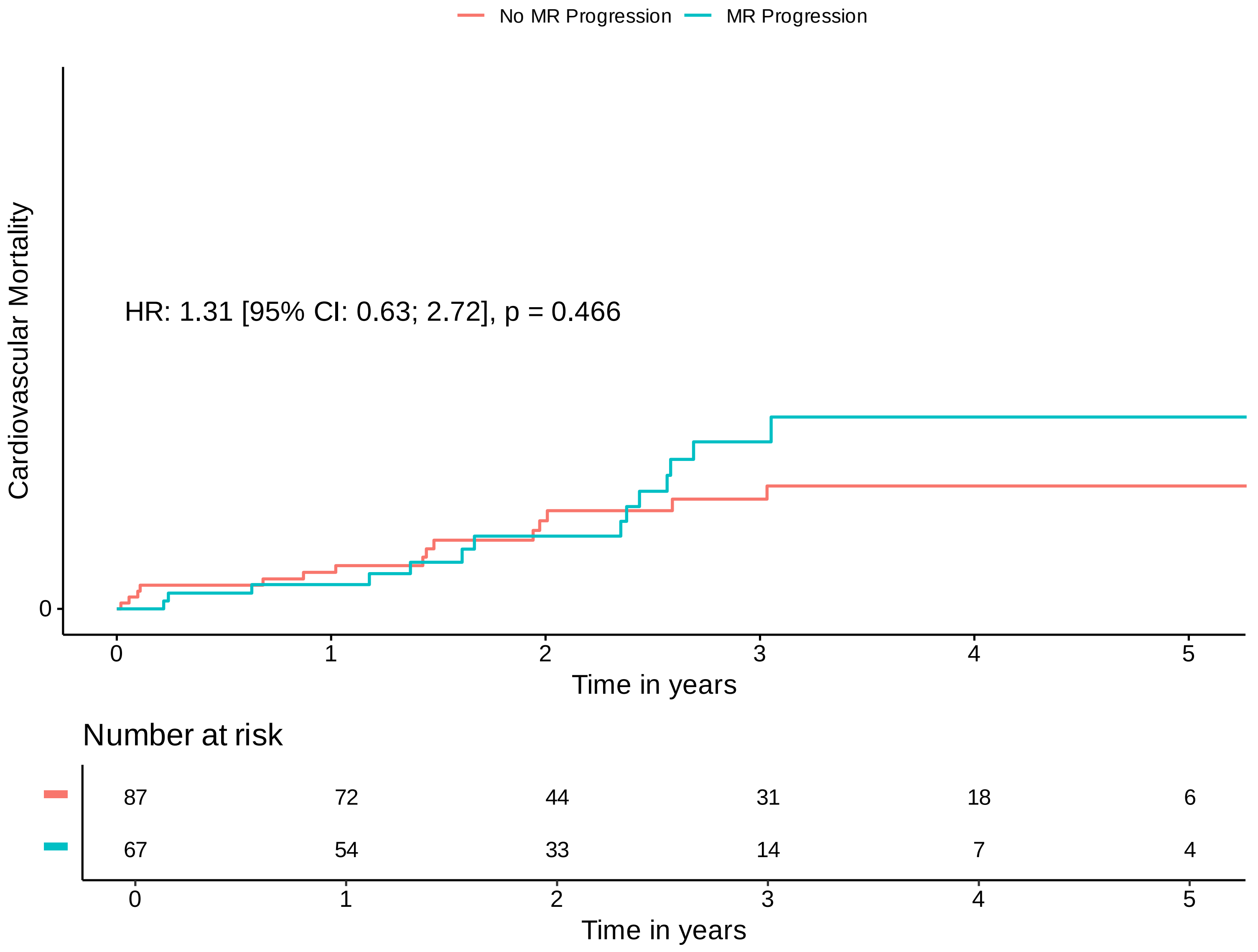

Figure S4 – Kaplan Meier curve for stroke with Cox regression analysis on mitral regurgitation progression

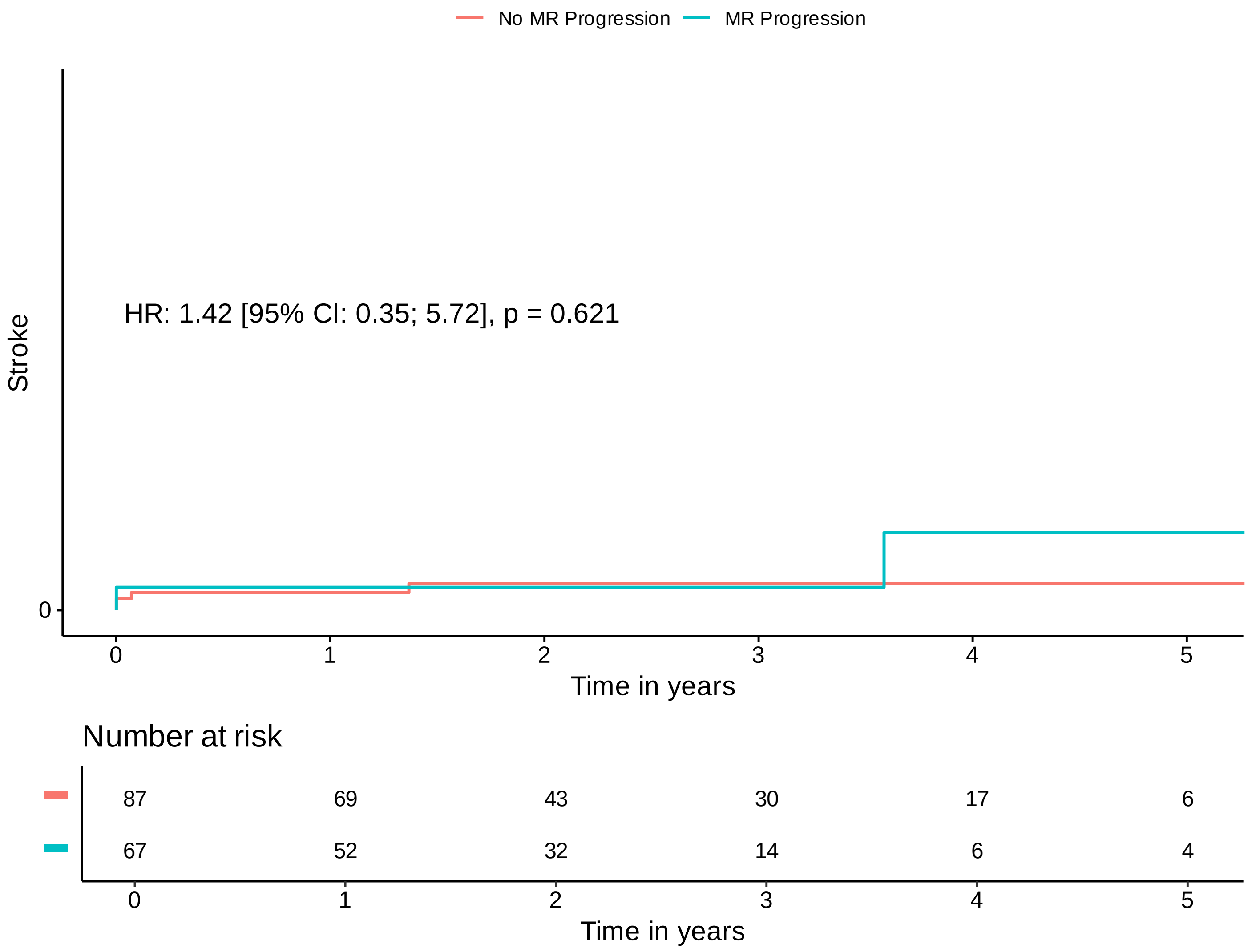

Supplement: Supplementary file 1 [file medicina-61-00686-s001.zip › medicina-3543343-supplementary.pdf]
